# Supplementary material for: Timekeeping in the hindbrain: a multi-oscillatory circadian centre in the mouse dorsal vagal complex
Source: Commun Biol. 2020 May 8;3:225. doi: 10.1038/s42003-020-0960-y (PMC7210107; doi:10.1038/s42003-020-0960-y)
Supplement: Supplementary file 1 — Reporting Summary [file 42003_2020_960_MOESM1_ESM.pdf]

nature research

Corresponding author(s): Chrobok, Northeast, Myung, Cunningham, Petit, Piggins

Last updated by author(s): Mar 20, 2020

Reporting Summary

Nature Research wishes to improve the reproducibility of the work that we publish. This form provides structure for consistency and transparency in reporting. For further information on Nature Research policies, see Authors & Referees and the Editorial Policy Checklist.

Statistics

For all statistical analyses, confirm that the following items are present in the figure legend, table legend, main text, or Methods section.

n/a	Confirmed	
☐	☒	The exact sample size (*n*) for each experimental group/condition, given as a discrete number and unit of measurement
☐	☒	A statement on whether measurements were taken from distinct samples or whether the same sample was measured repeatedly
☐	☒	The statistical test(s) used AND whether they are one- or two-sided<br>*Only common tests should be described solely by name; describe more complex techniques in the Methods section.*
☒	☐	A description of all covariates tested
☒	☐	A description of any assumptions or corrections, such as tests of normality and adjustment for multiple comparisons
☐	☒	A full description of the statistical parameters including central tendency (e.g. means) or other basic estimates (e.g. regression coefficient) AND variation (e.g. standard deviation) or associated estimates of uncertainty (e.g. confidence intervals)
☒	☐	For null hypothesis testing, the test statistic (e.g. F, t, r) with confidence intervals, effect sizes, degrees of freedom and P value noted<br>*Give P values as exact values whenever suitable.*
☒	☐	For Bayesian analysis, information on the choice of priors and Markov chain Monte Carlo settings
☒	☐	For hierarchical and complex designs, identification of the appropriate level for tests and full reporting of outcomes
☒	☐	Estimates of effect sizes (e.g. Cohen's *d*, Pearson's *r*), indicating how they were calculated

Our web collection on statistics for biologists contains articles on many of the points above.

Software and code

Policy information about availability of computer code

Data collection	Electrophysiology: MC Rack 4.6.2 (Multichannel Systems, Germany; Bioluminescence: Hokawo 2.0 (Hammamatsu, Japan); Confocal: LAS (Leica, Germany), qPCR: Stepone Plus (Life Technologies).
Data analysis	ImageJ, Fiji, Spike2 8.11 (Cambridge Electronic Design, UK), Neuroexplorer 5, MC Data Toll 2.6.15, OrginPro9.1, GraphPad Prism 7, Mathematica, El Temps.

For manuscripts utilizing custom algorithms or software that are central to the research but not yet described in published literature, software must be made available to editors/reviewers. We strongly encourage code deposition in a community repository (e.g. GitHub). See the Nature Research guidelines for submitting code & software for further information.

Data

Policy information about availability of data

All manuscripts must include a data availability statement. This statement should provide the following information, where applicable:

- Accession codes, unique identifiers, or web links for publicly available datasets
- A list of figures that have associated raw data
- A description of any restrictions on data availability

Data that support these findings are available on reasonable request from the corresponding author.

Field-specific reporting

Please select the one below that is the best fit for your research. If you are not sure, read the appropriate sections before making your selection.

☒ Life sciences ☐ Behavioural & social sciences ☐ Ecological, evolutionary & environmental sciences

Life sciences study design

All studies must disclose on these points even when the disclosure is negative.

Sample size	No Sample Size calculations were done. For bioluminescence experiments, n number sample size was based on previous experience (Guilding et al., 2009; 2010) and known coefficient of variation in these experiments showed that similar sample sizes were sufficient.
Data exclusions	Data exclusions, if done, were based on outlier identification in GraphPad Prism 7.
Replication	Immunohistochemical experiments were performed twice, in LD and DD conditions, with findings reproduced. Where possible, electrophysiological experiments were replicated.
Randomization	Randomization was not required as we were not comparing between genotypes of differences in compounds. Efforts were taken to ensure mice of similar ages and sex were used throughout the comparison groups.
Blinding	Image analysis was blinded and performed independently by two investigators. Sigma

Reporting for specific materials, systems and methods

We require information from authors about some types of materials, experimental systems and methods used in many studies. Here, indicate whether each material, system or method listed is relevant to your study. If you are not sure if a list item applies to your research, read the appropriate section before selecting a response.

Materials & experimental systems

n/a	Involved in the study	
☐	☒	Antibodies
☒	☐	Eukaryotic cell lines
☒	☐	Palaeontology
☐	☒	Animals and other organisms
☒	☐	Human research participants
☒	☐	Clinical data

Methods

n/a	Involved in the study	
☒	☐	ChIP-seq
☒	☐	Flow cytometry
☒	☐	MRI-based neuroimaging

Antibodies

Antibodies used	Sigma G6171 Monoclonal G-A-5; Abcam ab24525 Polyclonal igY fraction; Jackson Immunoresearch 715-165-150 and 703-545-155 both Polyclonal Whole IgG.
Validation	Commercially validated.

Animals and other organisms

Policy information about studies involving animals; ARRIVE guidelines recommended for reporting animal research

Laboratory animals	Mouse. mPer2Luc knockin mice, mice from Charles River, Kent, aged 10-12weeks, all males. mPer2::Luciferase knock-in male mice, backcrossed to C57BL6J for > 8 generations. 10-20 weeks of age.
Wild animals	*Provide details on animals observed in or captured in the field; report species, sex and age where possible. Describe how animals were caught and transported and what happened to captive animals after the study (if killed, explain why and describe method; if released, say where and when) OR state that the study did not involve wild animals.*
Field-collected samples	*For laboratory work with field-collected samples, describe all relevant parameters such as housing, maintenance, temperature, photoperiod and end-of-experiment protocol OR state that the study did not involve samples collected from the field.*
Ethics oversight	All experiments involving animals were carried out with approval by the Research Ethics committee of the University of Manchester and in keeping with the UK Animal (Scientific Procedures) Act 1986.

Note that full information on the approval of the study protocol must also be provided in the manuscript.
