# Supplementary material for: Timekeeping in the hindbrain: a multi-oscillatory circadian centre in the mouse dorsal vagal complex
Source: Commun Biol. 2020 May 8;3:225. doi: 10.1038/s42003-020-0960-y (PMC7210107; doi:10.1038/s42003-020-0960-y)
Supplement: Supplementary file 2 — Supplementary Information [file 42003_2020_960_MOESM2_ESM.pdf]

Supplementary Figure 1

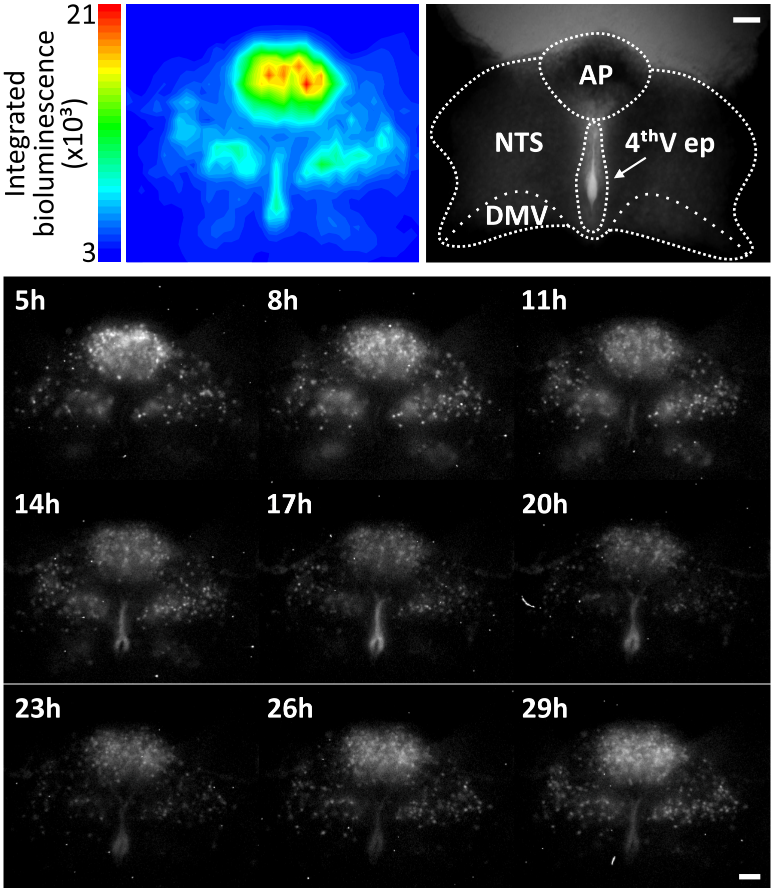

Supplementary Figure 1. Spatio-temporal localisation of the PER2::LUC bioluminescence within the DVC. (*top panel*) Heatmap representing the maximal PER2::LUC expression over 24h in one representative live coronal brainstem slice with delineation of anatomical structures overlaid: AP - area postrema; NTS - nucleus of the solitary tract; DMV - dorsal motor vagus; 4th V ep - fourth ventricle ependymal cell layer. (*bottom panel*) Raw images showing the change in PER2::LUC expression every 3h. Horizontal white bar depicts 100 µm.

Supplementary Figure 2

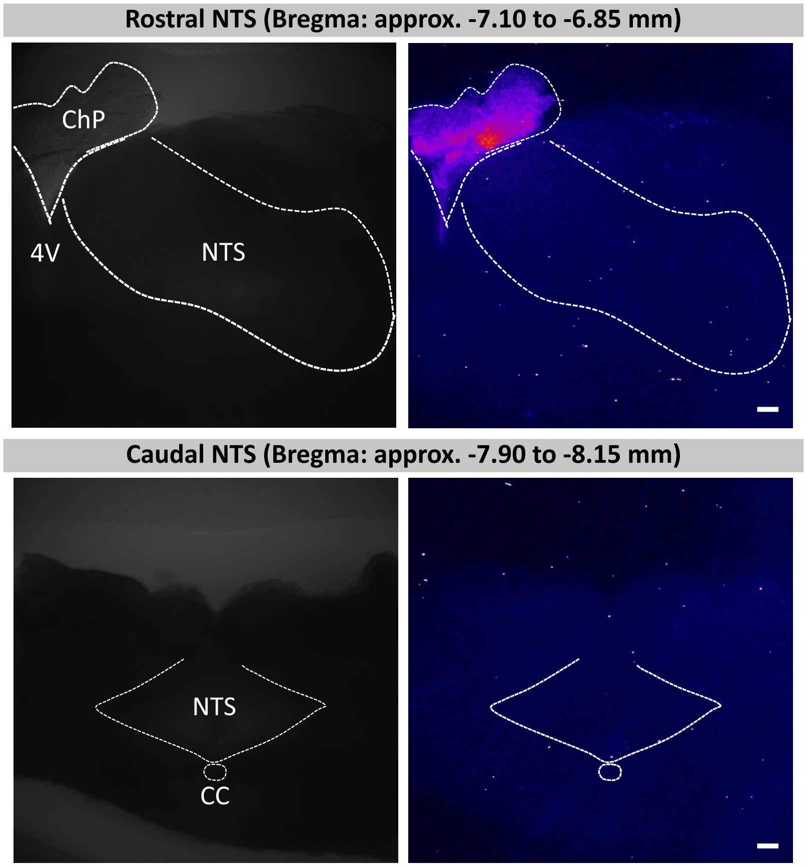

Supplementary Figure 2. Absence of PER2:LUC signal in the rostral and caudal NTS. Bright field photographs (*left*) and corresponding false-coloured bioluminescence images (*right*) with the anatomical representations of brainstem centres: 4V – 4th ventricle, CC – central canal, ChP – choroid plexus of the 4th ventricle, NTS – nucleus of the solitary tract. Note the high bioluminescence of ChP and lack of signal in NTS sections that do not contain the AP. White bars depict 100 µm.

Supplementary Figure 3

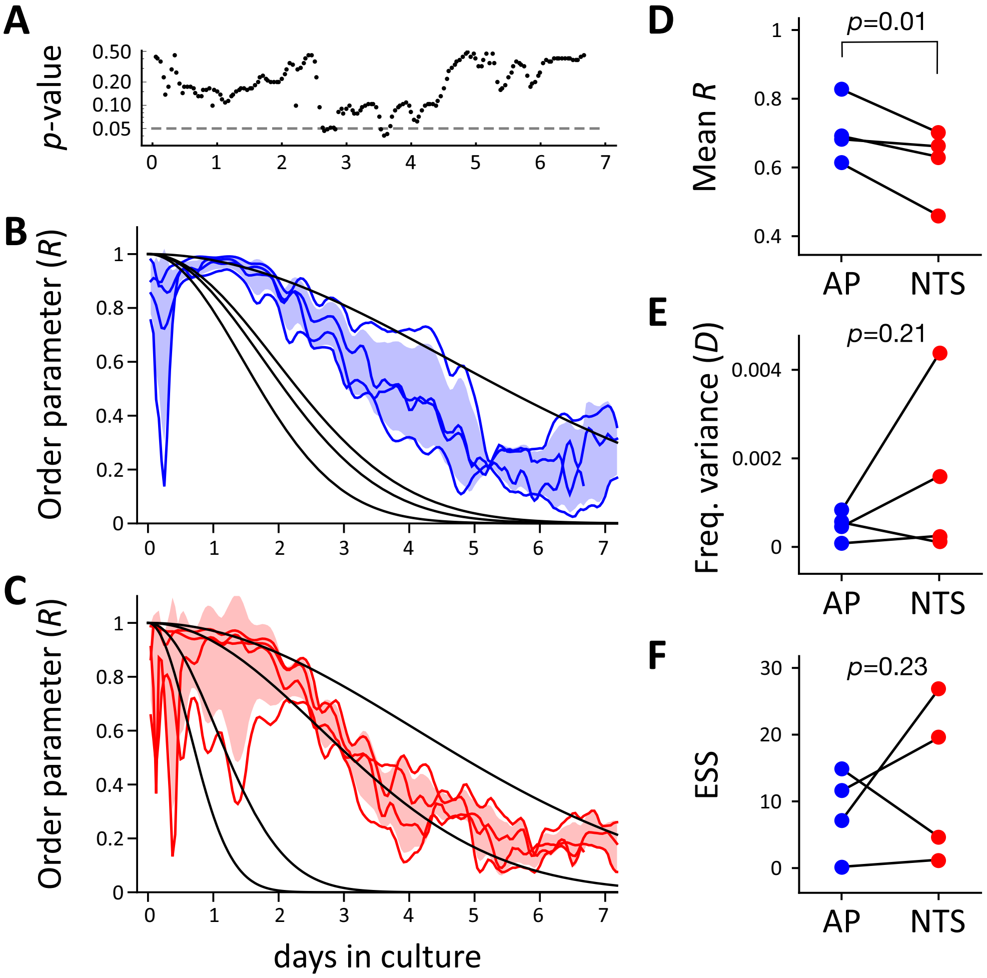

Supplementary Figure 3. Detuning from initial synchronised state reveals dynamic relation between the AP and NTS. A. AP-NTS differences in detuning dynamics are pronounced after 2.5-4.5 days *in vitro* (one-sided Welch's *t*-test was used due to unequal variance). **B.** Temporal changes in the degree of synchronisation for 4 control slices were characterised using Kuramoto's order parameter *R*. Solid blue lines indicate *R* for the AP in each slice and the shade indicates the standard deviation among slices. Solid black lines indicate predicted detuning under zero-

coupling assumption. **C.** Same as **B** for the NTS, indicated in red. **D.** Time-averages of **B** and **C**, for the first 5 days in culture. These reveal that the AP (*blue*) sustains synchronisation for longer than the NTS (*p=0.01; paired t-test). **E.** Variance of circadian frequency distribution estimated from the first 2 days' traces. The frequency variance was used to predict detuning under zero-coupling in **B** and **C**. There is a trend for the AP to have a narrower frequency distribution than the NTS, although the difference is not statistically significant (p=0.21, paired t-test). **F.** Error sum of squares (ESS) was estimated by finding the squared differences between the order parameter and predicted zero-coupling detuning. The ESS indicates the extent to which the observed order parameter deviates from the zero-coupling assumption, and hence indicates the presence of coupling. Although not significantly different (p=0.23, paired t-test), it implies that NTS synchronisation depends on the network coupling. In **D – F** each point indicates one slice culture. Data are expressed as mean ± SEM.

Supplementary Figure 4

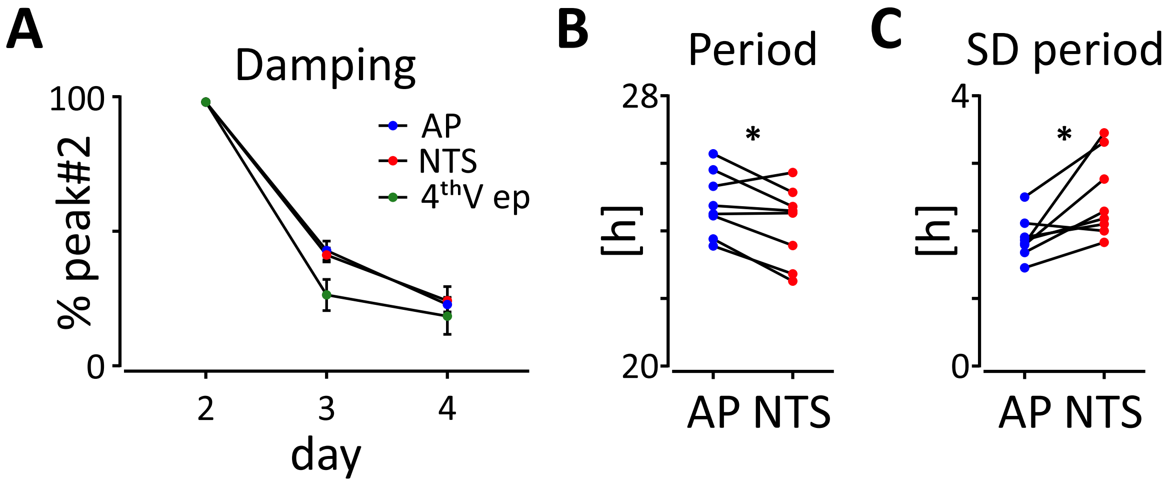

Supplementary Figure 4. Temporal properties of PER2::LUC rhythms in the DVC. A. Damping rate relative to the amplitude at peak 2 does not vary between the distinct DVC oscillators on the whole tissue level. **B & C.** NTS single cell oscillators are characterised by a shorter but more variable period than those in the AP (SD period; *$p<0.05$, $n=8$, *t*-tests). Data are expressed as mean ± SEM.

Supplementary Figure 5

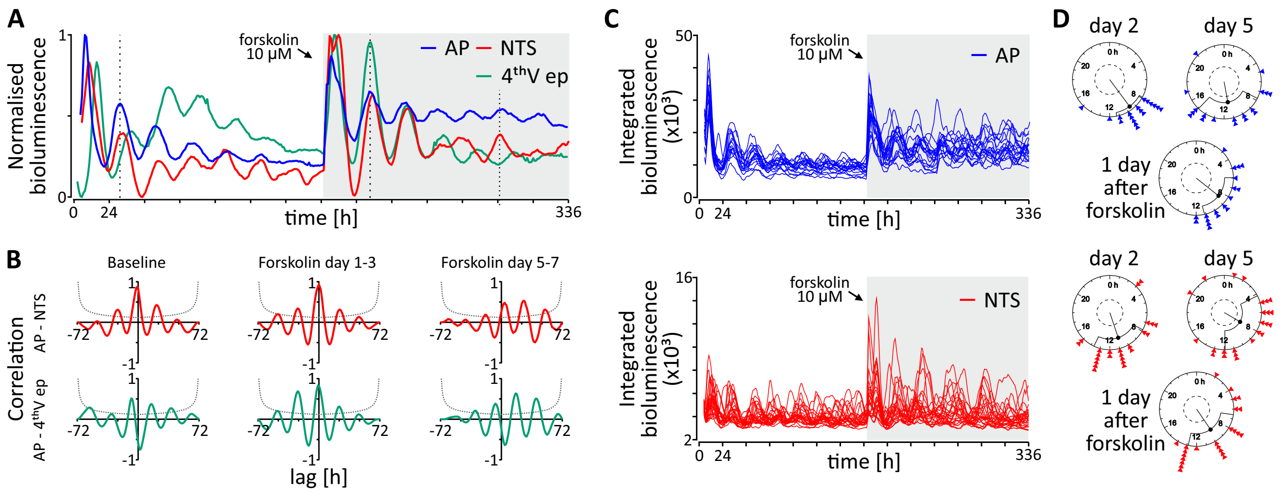

Supplementary Figure 5. Forskolin restores rhythmicity and transiently phase-aligns brainstem oscillators. A. Bioluminescence traces for whole brainstem area rhythms for 7 days before and after 10 µM forskolin treatment, with dotted lines representing the time at which the corresponding cross-correlograms in **B** were taken. **B.** Cross-correlograms showing the representative phase relationship for the NTS vs AP (*red*) and the 4thV ep vs AP (*green*) for baseline (*left panels*), days 1-2 (*middle panels*) and days 5-7 (*right panels*) post 10 µM forskolin treatment. **C.** Traces of putative single cell PER2::LUC over 14 days with 10 µM forskolin treatment at day 7. **D.** Rayleigh plots depicting phase clustering for the single cell bioluminescence traces in **C** for baseline day 2 and 5, and day 1 after 10 µM forskolin treatment.

Supplementary Figure 6

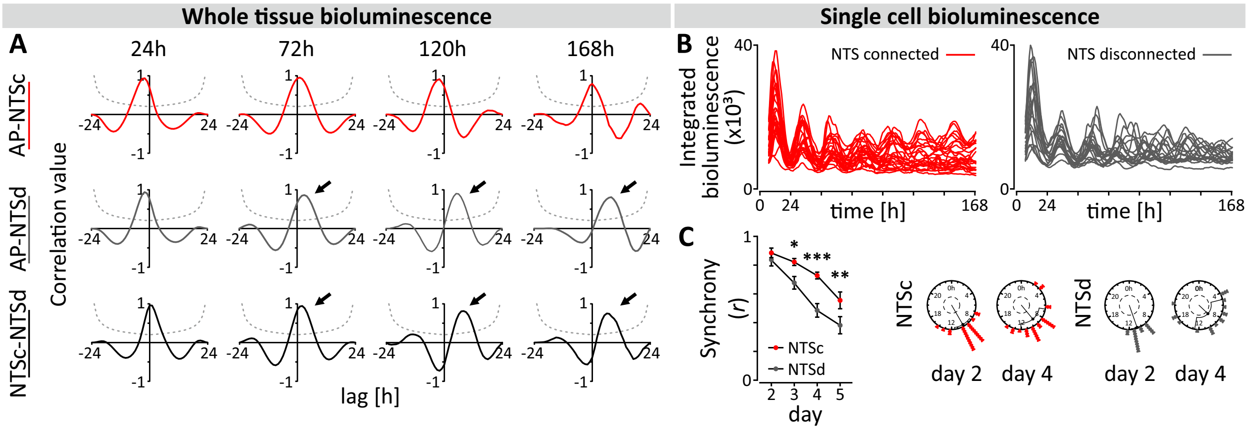

Supplementary Figure 6. Unilateral mechanical disconnection of the NTS from the AP affects its circadian phasing and synchrony. A. Stable phase relationship between the AP and the NTS connected to the AP (NTSc) throughout the whole recording is depicted at cross-correlograms (*in red*). Due to its shorter period, the phase of the disconnected NTS (NTSd) drifts from both the AP (*in grey*) and NTSc (*black cross-correlograms*), as highlighted by black arrows. **B.** Example traces of PER2::LUC bioluminescence at the putative single cell level over 7 days in culture, traced for each ROI individually (NTSc – *red*, NTSd – *grey*). **C.** Rayleigh *r* values showing synchrony amongst single cell oscillators from day 2 to 5 with example Rayleigh plots of day 2 and day 4 in culture. NTSd was significantly desynchronised after 3 days in culture compared to its connected counterpart (day 3: *p=0.0201; day 4: ***p=0.0004; day 5: **p=0.0060, Sidak's tests). Data are expressed as mean ± SEM.

Supplementary Figure 7

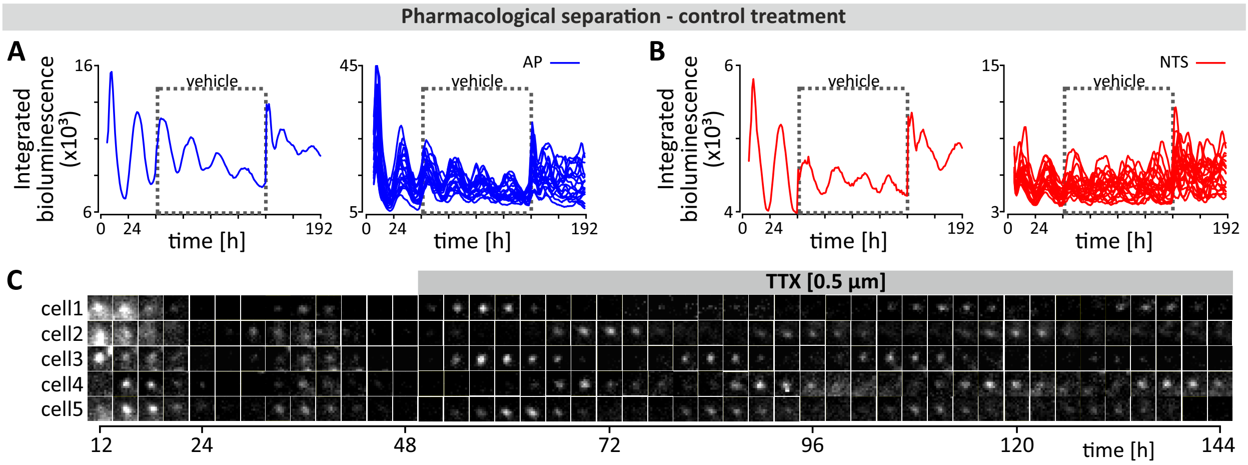

Supplementary Figure 7. Results of the treatment procedures on the PER2::LUC bioluminescence imagining of DVC explants. A & B. Example PER2::LUC bioluminescence trace showing the vehicle (water) application into after two days in culture (AP – *blue*, NTS – *red*) at the whole tissue and single cell bioluminescence level. Dotted boxes mark the period of treatment (4 days). Note, that this control procedure did not alter damping in bioluminescence nor cause desynchrony of single cell oscillators. **C.** Example tracing for five independent single cell oscillators in the NTS, sampled each 3h (for clarity). TTX treatment delineated by the grey box. Note, that after drug application, the phase of single cells uncoupled.

Supplementary Figure 8

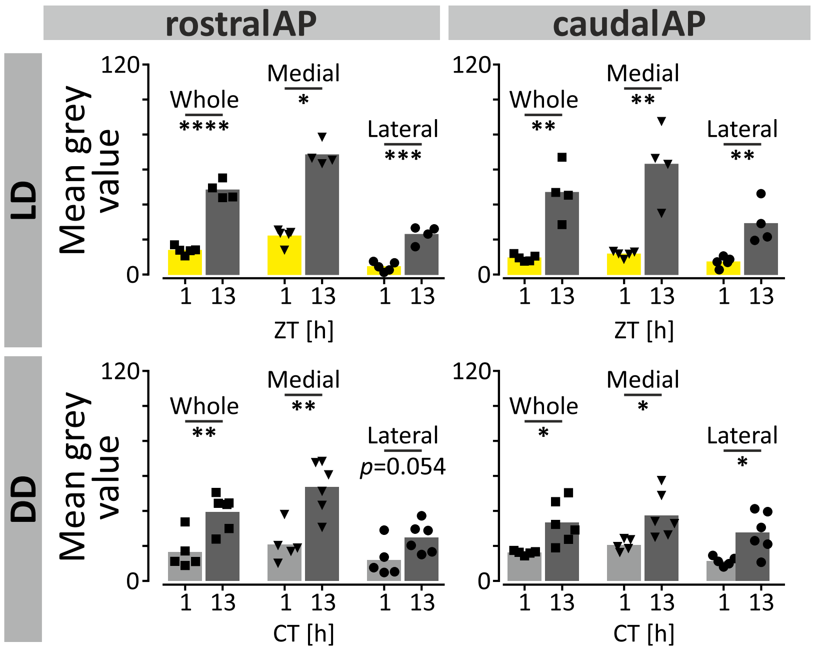

Supplementary Figure 8. Evans Blue (EB) penetration to the NTS in the rostral and caudal divisions of the AP increases at night. Intensity of EB staining in the whole, medial and lateral areas of the NTS for each rostral and caudal sample slice during LD (*top row*) and DD (*bottom row*) (**p*<0.05, ***p*<0.01, ****p*<0.001, *****p*<0.0001, *t*-tests or Mann-Whitney tests).

Supplementary Figure 9

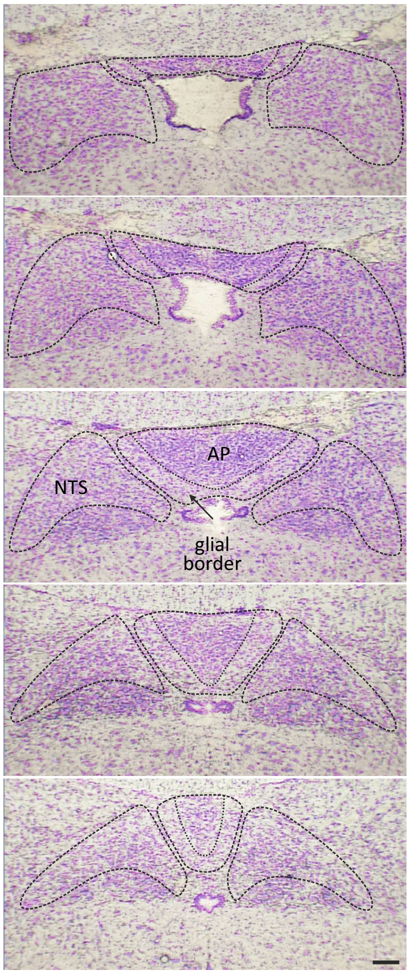

Supplementary Figure 9. Coronal sections of the DVC for laser-capture RNA extraction. Annotations of the ROIs on the Nissl stained tissue show (1) the NTS and (2) AP with the glial border (collected together). Slices are presented from the rostral to caudal extent of the DVC. Black bar depicts 100 µm.

Supplementary Figure 10

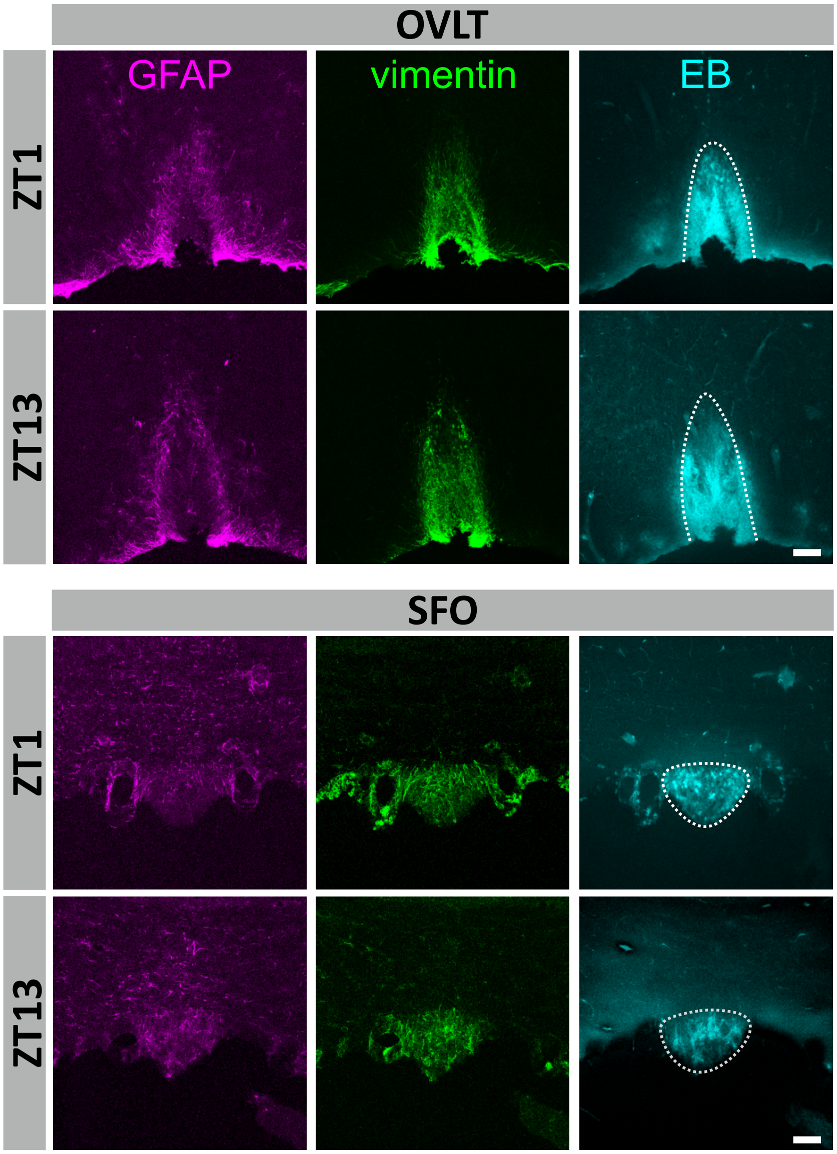

Supplementary Figure 10. Evans Blue (EB) is contained in the vascular organ of the lamina terminalis (OVLT) and subfornical organ (SFO) in the day and night. Glial markers for circumventricular organs (GFAP - *magenta*, vimentin - *green*) were used to define anatomical borders (broken lines) of the OVLT and SFO for the assessment of EB staining (*cyan*). Note the lack of EB penetration into brain parenchyma at either time point. White bars depict 100 µm.
