# Supplementary material for: Timekeeping in the hindbrain: a multi-oscillatory circadian centre in the mouse dorsal vagal complex
Source: Commun Biol. 2020 May 8;3:225. doi: 10.1038/s42003-020-0960-y (PMC7210107; doi:10.1038/s42003-020-0960-y)
Supplement: Supplementary file 3 — Description of Additional Supplementary Files [file 42003_2020_960_MOESM3_ESM.pdf]

Additional Supplementary File

File name: Supplementary Data 1

Description: Source data for Figures 1 to 6.
